# Long-Term Giant Hogweed Invasion Contributes to the Structural Changes of Soil Nematofauna

**DOI:** 10.3390/plants10102103

**Published:** 2021-10-04

**Authors:** Marek Renčo, Jana Jurová, Erika Gömöryová, Andrea Čerevková

**Affiliations:** 1 Institute of Parasitology, Slovak Academy of Sciences, Hlinkova 3, 040 01 Košice, Slovakia; jjurova@saske.sk (J.J.); cerev@saske.sk (A.Č.); 2 Faculty of Forestry, Technical University in Zvolen, T.G. Masaryka 24, 960 53 Zvolen, Slovakia; gomoryova@tuzvo.sk

**Keywords:** *Heracleum*, invasive species, soil nematoda, grassland, diversity

## Abstract

*Heracleum mantegazzianum* (giant hogweed) is the largest central European forb, naturalized or invasive in many European countries. The impacts of its colonization of native habitats on soil mesofauna groups are unfortunately obscure. This study assessed the effect of giant hogweed invasion on the communities of plants and soil nematodes in the riparian habitat. We found that invasion by *H. mantegazzianum* increased soil pH, decreased carbon and nitrogen content, reduced the number and coverage of the native plant species, and influenced nematode communities and their structures. Nematode species number was significantly lower in invaded than uninvaded plots, but nematode species diversity was not affected by invasion throughout the whole study. Total nematode abundance slightly increased under giant hogweed, while total nematode biomass did not differ between the invaded and uninvaded plots. The higher abundance of bacterivores and fungivores but lower number of omnivorous nematodes well represented the negative impact of giant hogweed invasion on soil food webs, supported by low values of all maturity indices or channel index. The hogweed invaded plots contained higher abundance of plant parasitic nematodes, mainly *Paratylenchus microdorus*. Our results thus indicate that invasion by *H. mantegazzianum* influences several nematode communities’ parameters while others remain unaffected by invasion.

## 1. Introduction

The invasion of non-native species is becoming one of the major threats for global biodiversity [1]. Compilation of a global dataset of regional first reports of alien species by [2] revealed that 37% of all first records of invasive species were reported within the period of 1970–2014. Among these organisms, an important position is occupied by invasive plants, which in general have several biological traits that allow them to win the competition with native species. For example, rapid juvenile germination and next growth to reproductive adult reduces generation time and allows the next generation to be produced quickly [3]. The high seed production facilitates the establishment of large numbers of individuals on site [4] and reduces the availability of sunlight and other resources necessary for growth of native species; therefore, vegetative (clonal) growth allows many invasive species to reproduce and survive in the absence of suitable pollinators and the ability to effectively and quickly cover a site [5]. Due to plasticity, invasive species may grow and create monospecific communities in a range of biotic conditions [6]. However numerous studies have shown the divergent impacts of non-native plant species on ecosystems and the contrasting effects of biotic and abiotic factors on the dynamics of non-native species. This is hindering the emergence of a unified theory of biological invasions [7], but one theory claims that a plant community becomes more susceptible to invasion whenever there is an increase in the amount of unused resources [8]. This theory rests on the simple assumption that an invading species must have access to available resources (e.g., light, nutrients, and water) and that a species will enjoy greater success in invading a community if it does not encounter intense competition for these resources from the resident species. This assumption is grounded in the theory that competition intensity should be inversely correlated with the amount of unused resources [9].

The genus *Heracleum* L. contains more than 120 species and is one of the largest genera of the family Apiaceae. Some of the large species of the genus, called “large, tall, or giant hogweeds” have become invasive or naturalized in many European countries, namely *Heracleum mantegazzianum* Sommier & Levier, *H. sosnowskyi* Manden. and *H. persicum* Desg. Ex. Fischer [10]. The study species, *Heracleum mantegazzianum* (*Hm*), is a biennial or perennial monocarp tall herb, native to the southern slopes of the Western Greater Caucasus, where it grows in meadow communities, forest clearings, and forest margins in the upper forest belt [11]. It was introduced into Europe as a garden ornamental in 1817, when it appeared on the seed list at Kew Botanic Gardens, London. In 1828, the first population was recorded growing wild in Cambridgeshire, England. Ranked according to the date of introduction, the UK was followed during the second half of the 20th century by the Netherlands, Switzerland, Germany, Ireland, Denmark, and the Czech Republic [10,12,13,14]. In the Slovak Republic, the first report on giant hogweed was published by [15]. Since 2000, several localities with sporadic occurrence of *H. mantegazzianum* through the country have been found such as Javorníky, Kysuce, Low Tatras, High Tatras, Low Fatra, and Chočské vrchy [16,17,18,19,20]. The most distinctive characteristic of *Hm* is its size, where individuals can achieve heights up to 3–5 m, thus belonging to the tallest and largest herbs in Europe. Giant hogweed has a good competitive ability and a high seed production and therefore has become an aggressive invasive weed causing many problems. Pyšek [21] compared the rate of invasion of *Hm* in Europe at three spatial scales (continental, regional, and local). The invasion was slowest at the continental scale (62 years) and did not differ significantly between regional (16 years) and local (22 years) scales. This indicates that there are two different mechanisms of spread acting together in this system, namely human influences and natural spread, and the relative influence of these mechanisms appears to change in an inverse proportion from the largest to the smallest scale. At the local scale, under suitable habitat conditions, the process is driven by biological traits of the species related to dispersal. At the continental and regional scales, humans play a crucial role in the invasion of *H. mantegazzianum* by planting it as a garden ornamental [21].

It is well known that *Hm* has serious health implications for humans due to phyto-photo-dermatitis caused by furocoumarins (syn. furanocoumarins) contained in the sap of the plant [22]. However, unlike many other invasive species, the majority of which do not form large populations in new areas, *Hm* usually occurs as a dominant species of invaded communities in many different habitat types [23]. Therefore, its invasion causes havoc in native plant communities and decreases species diversity and/or species number [24]. A study by [25] revealed that invasion by *Hm* decreased soil organic matter weight, C and N contents, and C mineralization, while [23] observed that invasion increased soil pH, soil conductivity, and P content. In contrast, the presence of *Hm* positively affects the behavior of native plant pollinator [26], ant activity, and the number of myrmecophil aphids [27], while it did not affect soil microbial communities [23]. However, the behavior and response of large groups of small soil inhabitants (e.g., nematodes, to the incidence of *Hm* have never been studied).

Among the various soil inhabitants, nematodes represent one of the most abundant and diverse metazoan groups in terrestrial ecosystems [28]. They cover several trophic levels by feeding on algae and plants, on bacteria and fungi, or on soil animals (in particular other nematodes), thus being a central element of the soil food web [29]. This makes them particularly suitable for studying global change effects on different trophic levels within the same faunistic group. All nematodes can be referred to two main reproductive strategies: (1) *K* strategists do best in stable environments, are larger, and have long life cycles with small population increases; and (2) *r* strategists increase rapidly under favorable conditions, are quite small, and have short life cycles and high reproductivity [30]. Thus, they are useful bioindicators with their functional shifts providing valuable information on the state of an ecosystem, allowing for inferences to other biotic groups and soil health [31]. Representation of nematode species/genera within community or abundance of trophic groups include colonizer–persister values of taxa allow for the calculation of various ecological indices and ratios, parameters that suggest an easier functional interpretation in relation to disturbance.

Wolfe and Klironomos [32] proposed three linkages that are directly impacted by invasive species: (i) plant community composition and ecosystem processes; (ii) plant community composition and soil community composition; and (iii) soil community composition and ecosystem processes. Nevertheless, impacts of alien plant species as well as answer of native above and/or belowground soil biota in natural habitats where invasion has taken place are difficult to predict because (i) the places where invasion will take place are unpredictable, therefore, (ii) the data about the communities of native organisms in places before invasion are unknown, and (iii) the traits of the invading species, which are variable and many times new for ecosystems (i.e., individual size of biomass, root area, leaf area, presence of perennial tissue, clonal growth, salinization, or ability to fix nitrogen) [33]. Therefore, we can only compare the community structure of nematodes inhabiting *Hm* invaded plots with communities in uninvaded plots located nearby, hoping that both plots had similar plant and nematode composition before the *Hm* entry. Several previous studies by our research group on the related species *H. sosnowskyi* carried out in various types of habitats where invasion took place in Lithuania [34], Poland [35], and Russia [36], however, revealed significant shifts in plant species composition, which subsequently modified nematode assemblages and trophic structures. The present study (1) investigated nematode species diversity, abundance, nematode trophic group composition, and nematode food web characteristics in the *H. mantegazzianum* invaded and related uninvaded riverbank grassland, and (2) evaluated whether long-term *H. mantegazzianum* invasion affected nematode communities. We hypothesize that the impact of giant hogweed invasion on nematode communities is generally similar to its botanical related species *H. sosnowskyi* due to changes in native plant species composition.

## 2. Results

### 2.1. Soil Properties

The soil properties we evaluated differed between *H. mantegazzianum* invaded and UNV not-invaded plots as well as sampling date. The highest soil moisture was recorded in the summer sampling date at both plots, but without significant difference in the spring and autumn season. Soil pH was significantly higher in HMG than in UNV (*p* < 0.05) during the whole study. In contrast, available nitrogen and organic carbon contents were significantly lower in HMG plots than in UNV (*p* < 0.05). The C/N ratio did not differ statistically between the HMG and UNV plots during the whole study (Table 1).

Factorial analysis of variance also revealed that two main factors (sampling date and invasion status) as well as their interactions had a significant effect on all physico-chemical parameters analyzed including C/N ratio (*p* < 0.05; *p* < 0.01) (Table S1).

### 2.2. Plant–Community Analysis

**Figure 1:**
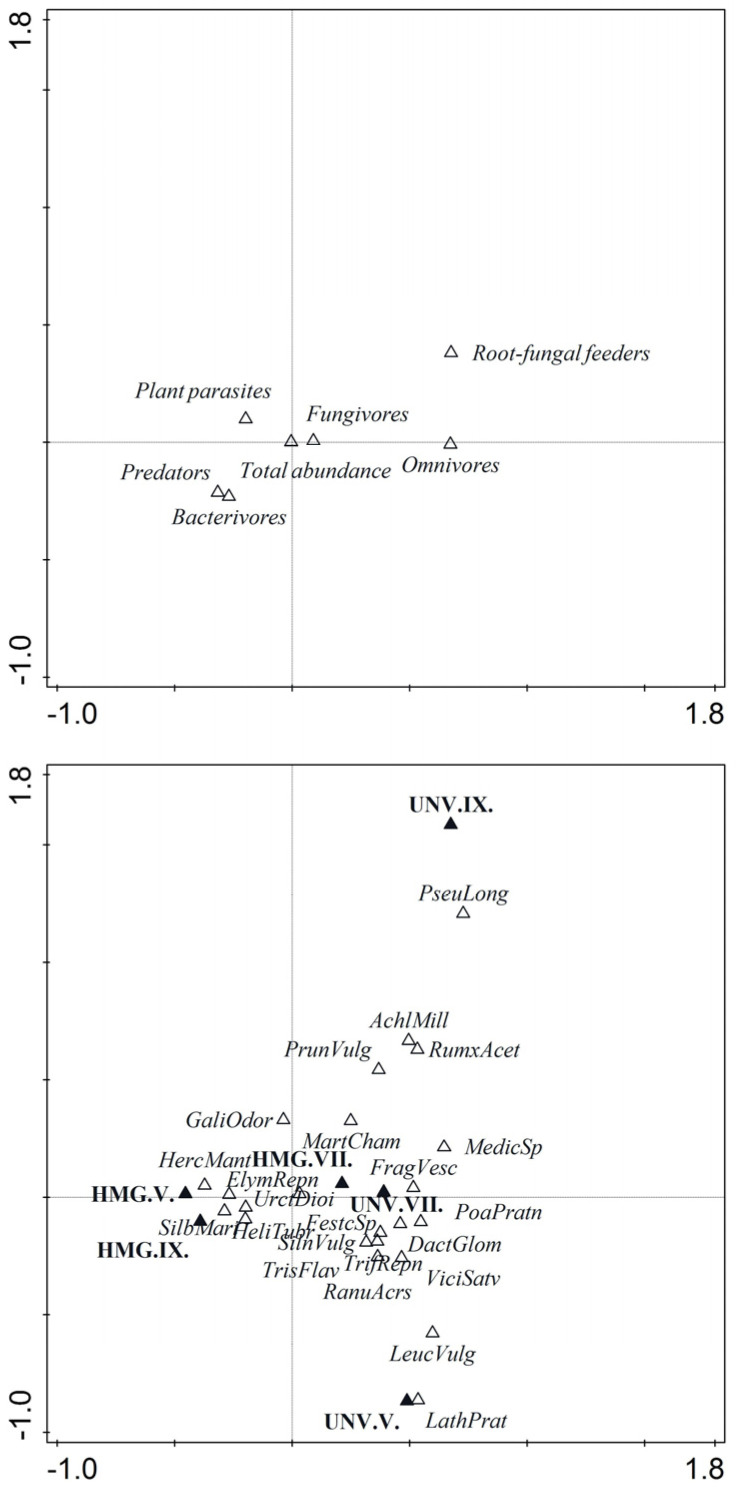
Biplot based on co-correspondence analysis illustrating the nematode trophic groups (**A**) and main plant species (**B**) common in *Heracleum mantegazzianum* invaded (HMG) and non-invaded (UNV) areas, 15.67% of the total variance of each dataset. Correlation coefficients between nematode-derived and plant-derived site scores of the first three axes of symmetry corresponded to the canonical analysis (axis 1: 0.6769, *λ*1-0.121, *p*-0.0320, axis 2: 0.6842). Symbols represent soil samples collected in May (V), July (VII), and September (IX) of the two vegetation seasons. Abbreviations used in panel (**B**): *AchlMill—Achillea milleofolium*; *DactGlom—Dactylis glomerata*; *ElymRepn—Elymus repens*; *FestcSp—Festuca* sp. *FragVesc—Fragaria vesca*; *GaliOdor*—*Galium odoratum*; *HeliTubr—Heliantus tuberosus*; *HercMant—Heracleum mantegazzianum*; *LathPrat—Lathyrum pratensis*; *LeucVulg*—*Leucanthemum vulgare*; *MatrCham*—*Matricaria chamomilla*; *MedicSp*—*Medicago* sp.; *PoaPratn*—*Poa pratensis*; *PrunVulg*—*Prunella vulgaris*; *PseuLong*—*Pseudoly simachion longifolium*; *RanuAcrs*—*Ranunculus acris*; *RumxAcet*—*Rumex acetosella*; *SilnVulg*—*Silene vulgaris*; *SilbMari*—*Silybum marianum*, *TrifRepn*—*Trifolium repens*; *TrisFlav*—*Trisetum flavescens*; *UrtcDioi*—*Urtica dioica*; *ViciSatv*—*Vicia sativa*.

The HMG and UNV plots were comparable regarding their herbaceous cover. The Co-CA was performed separately for each sampling date, but together for the two vegetation seasons indicated that *Hm* invasion significantly negatively affected the coverage of the native plant species throughout the study (Figure 1). Grasses such as *Dactylis glomerata* L., *Poa pratensis* L., *Trisetum flavescens* L., *Festuca* sp. or herbs such as *Silene vulgaris* (Moench) Garcke, *Vicia sativa* L. and *Matricaria chamomilla* L. were dominant in UNV, but absent in the HMG plots (Table 2). The dense vegetation coverage of *H. mantegazzianum* provided suitable conditions for *Urtica dioica*, *Galium odoratum*, or *Heliantus tuberosus*, which prevailed mainly in the autumn sampling date. However, in general, most species of native vegetation recorded in UNV were not recorded in HMG.

### 2.3. Nematode Community Analysis

A total of 82 nematode species, belonging to 61 genera were identified in our study (Table 3). The mean number of species varied considerably, ranging from 36 to 45, however, only in the summer and autumn sampling dates were significantly lower numbers of species in HMG compared to UNV plots recorded (*p* < 0.05) (Table 4).

Nevertheless, the nematode species diversity in HMG plots was uniform to that in the UNV plots within all sampling dates. The mean nematode abundance ranged from 245 to 412 individuals per 100 g of soil. Except for the spring sampling date, the HMG and CON plots could not be significantly differentiated in terms of the nematode abundance (HSD, *p* < 0.05), but the number of individuals was slightly higher in HMG than in UNV during the whole study.

Spearman’s rank correlations identified a negative relation between nematode abundance, number of species, and soil acidity (*p* < 0.05; *p* < 0.01) (Table S2).

Among the nematode trophic groups, in the study plots, the most diverse were bacterivores represented by 26 species, followed by plant parasites (17 species), omnivores (15 species), root-fungal feeders (nine species), fungivores (eight species), and predators (seven species) (Table 3). An HSD test identified significant differences in the abundance of bacterivorous nematodes between HMG and UNV throughout the whole study (*p* < 0.05) (Table 4). Among them, mainly abundant were species such as *Alaimus primitivus*, *Cephalobus persegnis*, *Eucephalobus oxyuroides*, *E. mucronatus*, or genus *Rhabditis* (Table 4). Similarly, fungivores were more abundant in HMG than in the UNV, mainly *Aphelenchoides parietinus*, *Aphelenchus avenae,* and *Diptherophora communis*, however, a significant difference only in the spring sampling date was recorded (HSD, *p* < 0.05). In contrast, omnivores (*Aporcelaimellus obtusicaudatus*, *Dorylaimus bryophilus*, *Pungentus silvestris*) were more abundant in UNV, while a significant decline in their mean number was not found. An HSD test of mean abundance of predators identified significant differences between HMG and UNV in the spring sampling date (*Discolaimoides bulbiferus*) (*p* < 0.05) as well as the obligate plant parasite (*Paratylenchus microdorus*), but differences were not significant (Table 4). Nevertheless, a factorial analysis of variance identified a significant main interaction between invasion status and the abundances of plant parasites and root fungal feeders (both *p* < 0.01) as well as bacterivores (*p* < 0.05), while season had an impact on the abundances of predators and omnivores (both *p* < 0.01) and bacterivores (both *p* < 0.05) (Table S3). The interaction of all three factors (invasion status, season, and sampling date) significantly affected only the numbers of plant parasitic nematodes (*p* < 0.05).

Spearman’s rank correlations identified a negative relation between the number of plant parasites, number of root-fungal feeders, and soil acidity (*p* < 0.05; *p* < 0.01) (Table S2). In contrast, fungivores positively correlated with N content (*p* < 0.05), plant parasites with soil moisture (*p* < 0.05), while omnivores with soil moisture and C content (*p* < 0.05; *p* < 0.01) (Table S2).

Additionally, the Co-CA revealed that total nematode abundance, number of bacterivores, plant parasites, and predators were more strongly associated with the presence of giant hogweed, *U. dioica*, *H. tuberosus*, and *G. odoratum* species in the HMG plots than with native species in the UNV plots (Figure 1). In contrast, Co-CA indicated that root-fungal feeders and omnivores were more strongly associated with the native plant species (Figure 1).

### 2.4. Analysis of Nematode Food Webs

Mean values of the community indices and metabolic footprints for HMG and UNV plots are given in Table 4. The presence of *H. mantegazzianum* had a significant and negative effect on the values of all maturity indices (MI, MI2-5 PPI and *∑*MI), especially in the summer and autumn sampling dates as well as channel index during the whole seasons (*p* < 0.05). The enrichment index, which characterizes the intensity of nutrient enrichment and structure index, which characterizes the soil food web structure, were not significantly different between HMG and UNV.

However, enrichment and bacterivore footprints were, on average, significantly higher in HMG compared to the uninvaded control plots (*p* < 0.05; *p* < 0.01). In contrast, there were no significant differences for other metabolic footprints between the invaded and uninvaded plots (Table 4). Spearman’s rank correlations revealed negative interactions between PPI, nematode biomass, and soil acidity (*p* < 0.05; *p* < 0.001) and MI, *∑*MI, and C content (*p* < 0.05; *p* < 0.01), while MI and nematode biomass positively correlated with the C and N contents, respectively (*p* < 0.05; *p* < 0.01) (Table S2).

**Figure 2:**
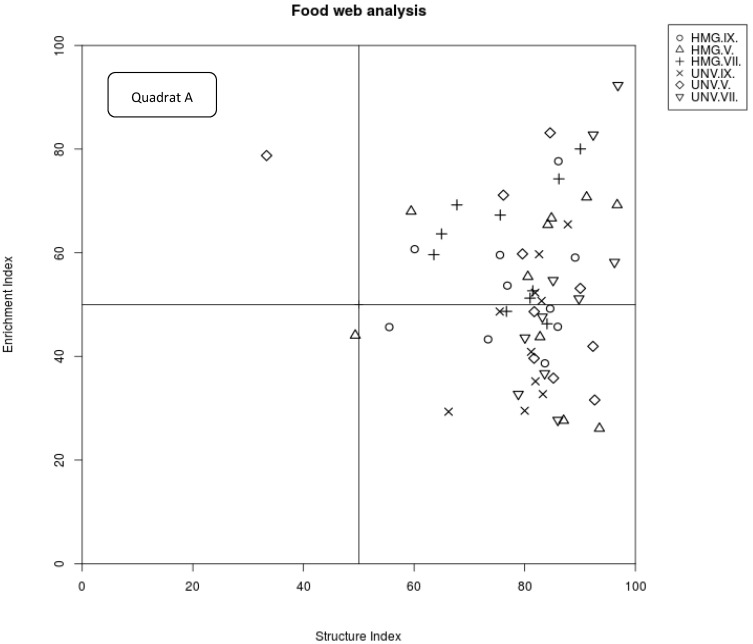
Faunal profiles of *Heracleum mantegazzianum* invaded (HMG) and uninvaded control (UNV) plots, representing the food web condition in relation to its structure (SI) and enrichment (EI), as indicated by the “weighted faunal analysis” following Ferris et al. (2001). Quadrat A should comprise nematode communities in an environment with a high degree of disturbance of the food web, N-enriched with low C/N ratio, and prevailing bacterial decomposition channel. Quadrat B represents an environment with a low to moderate degree of disturbance and maturing food web, N-enriched, and with balanced decomposition channel as well as low C/N ratio. Quadrat C represents an environment with an undisturbed and structured food web and relatively low primary production, fungal decomposition channel, and moderate to high C/N ratio (climax like). Quadrat D represents an environment with stressed and degraded food web condition, depleted with fungal decomposition channel, and high C/N ratio. Symbols represent soil samples collected in May (V), July (VII), and September (IX) of two vegetation seasons.

The enrichment index as an indicator of the level of primary enrichment and the structure index, which correlates with the degree of maturity of ecosystems, separated 63% samples of HMG to quadrat B, following the weighted faunal analysis by Ferris et al. (2001). This characterized the food web of HMG as maturing, the environment as low or moderately disturbed, and N-enriched with a balanced decomposition channel (Figure 2), which is consistent with the mean EI and SI values (Table 2). In contrast, the majority of samples collected in UNV are depicted in quadrat C, which represents an environment with an undisturbed and structured food web and fungal decomposition channel.

## 3. Discussion

Increasing the number of studies in recent years has demonstrated that individual plant species differently affect the communities of the soil food web they support [37,38,39], whereas several biodiversity experiments have revealed the adverse effect of plant species loss on soil Nematoda [40,41,42]. This suggest that shifts in plant community composition in the habitats where alien plant invasion have taken place could have substantial impacts on the communities’ structure of native soil nematodes.

Regarding *H. mantegazzianum*, only a few studies have investigated the impact of its invasion on surrounding living organisms (e.g., ants or aphids [27], honeybee [26], or interactions with phytophagous insects [43,44]). Unfortunately, to the best of our knowledge, no study has analyzed the impact of *Hm* invasion on any group of soil organisms or nematode communities. Several recent studies of our research group revealed that *Hm* botanically related invasive species *H. sosnowskyi* [34,35,36] or invasive species *Reynoutria japonica* [45] significantly influenced the structure of nematode communities and the abundance or number of species, indicating that *Hm* invasion of natural grasslands on river banks might alter soil nematode communities through changes in plant communities and soil properties [23,24,46].

Our study did reveal the negative impact of *Hm* invasion on the native plant species, mainly grasses such as *D. glomerata*, *P.* *pratensis*, *T. flavescens*, and *Festuca* sp., which disappeared in the invaded plots, confirming the results by [46] from the abandoned grassland habitat invaded by giant hogweed in Germany. This suggests that allelopathy (production of allelochemicals that inhibit the growth of native plants) could be one of the mechanisms of *Hm* invasion success, in agreement with current phylogenetic analysis by [47], who revealed that the majority of the 524 invasive plant species analyzed produced allelochemicals with the potential to negatively affect native plant performance. Nevertheless, the most frequent native tall forb in relevés with *Hm* was *U. dioica,* which is itself a strong and high-growing competitor (C-strategist) according to [48], or *H. tuberosus*, whose cover increased at the end of the vegetation cycle of giant hogweed (autumn).

Aside from the direct effect of invasive plants on native plant communities, they can also modify soil physico-chemical characteristics and nutrient cycling [49]. Our investigation carried out during two vegetation seasons revealed that *Hm* invasion considerably increased soil pH, in agreement with the findings of [23] from a long-time giant hogweed invaded protected forest in the Czech Republic. In contrast, soil nitrogen and carbon contents were significantly lower in HMG than UNV in the spring and summer sampling dates, contradicting the findings by [23,50] where the N and C contents did not change following *Hm* invasion, or in [51] from *R. japonica* invaded plots. The likely reason is that although hogweed biomass productivity compensated for the post-invasion decrease in native biomass, it did not increase the overall productivity at invaded sites [52].

As above-mentioned, to the best to our knowledge, nematode communities have never been studied in a natural habitat invaded by *Hm*; therefore, our data provide the first insights into the impact of its invasion on this abundant and ecologically important group of soil biota. The mean number of nematode species was significantly lower in HMG than in UNV (summer, autumn), and the mean nematode abundance increased under *Hm* while nematode diversity remained unaffected by invasions during the whole study. These results partially agree with our previous findings on related species *H. sosnowskyi* performed in central Lithuania [34], Poland [35], and the Moscow region of Russia [36] in various habitats. Long-term effects of *H. sosnowskyi* dominance were associated with decrease in nematode abundance, species, or genera number while nematode diversity remained unaffected by invasions. Invasion of several other invasive plant species (e.g., *Bromus tectorum* [53], *Spartina alterniflora* [54], *Solidago gigantea* [55], and *R. japonica* [45]) similarly negatively affected the abundance, species number or biomass of soil nematodes. In contrast, invasion by *Ambrosia trifida* and *Asclepias syriaca* did not negatively affect nematode abundance or species number [56,57]. This indicates that the impact of invasive plants on nematode species number and abundance depends on the invading plant species. Nematode species diversity was not affected by *Hm* invasion in our study, nevertheless, many species of native plants were absent in HMG. This finding corresponds with results from *H. sosnowskyi* invaded habitats in Latvia, Poland, and Russia [34,35,36].

A key component of soil biodiversity involved in soil fertility and plant productivity are bacterivores [58]. These bacterivores are mostly represented by protists and nematodes [59]. *Hm* invasion considerably increased the number of bacterivores, mainly some species such as *A. primitivus*, *C. persegnis*, *E. mucronatus*, *E. oxyuroides,* or genus *Rhabditis*. Similar data from habitats invaded by *H. sosnowskyi* were reported by [34,35]. The likely reason is that both *Heracleum* species produce a litter beneficial to bacterial populations, thus confirming previous findings by [23], where the composition of soil microbial communities was not altered by long-term *Hm* invasion. In contrast, invasive *F. japonica* provides a large amount of litter with high tannin concentrations to the soil, which decomposes slowly, therefore favoring fungi over bacteria [60] and supporting the results by [45], who observed a negative impact of *Reynoutria* invasion on the abundance of bacterial feeding nematodes.

Modifications of the soil environment by plant invasions can depend not only on the chemical composition of plant litter, but also on the release of secondary metabolites. *Hm* produces a great diversity of secondary compounds (e.g., flavonoids, terpenes, essential oils, furanocoumarins, and acetylenic compounds) [61]. Many furanocoumarins are toxic and are produced by plants as a defensive mechanism against various phytophagous pests, ranging from bacteria to insects and mammals [62]. Therefore, we could assume that they will also be toxic to plant parasites in the soil, which depend on the presence of higher plants with root systems serving as food sources. Contrary to expectations, plots with *Hm* contained moderately higher numbers of plant parasitic nematodes (mainly c-p2, e.g., *Paratylenchus*) than plots with diverse native vegetation, although not statistically significant. Together with bacterivores, these are responsible for the higher nematode abundance in the invaded than the uninvaded plots. Similarly, the genus *Paratylenchus* was more abundant in the forests invaded by *H. sosnowskyi* [36], or grasslands invaded by *S. gigantea* [55]. In contrast, [34] reported lower abundances of *Paratylenchus* nematodes in abandoned land invaded by *H. sosnowskyi*. Plant parasitic species with higher c-p value (3–5) such as *Helicotylenchus digonicus*, *Longidorus attenuates*, or *Xiphinema simile* were missing or less abundant in *Hm* plots, suggesting negative interactions of selected parasites with giant hogweed rhizosphere.

Omnivores and predators are considered as ‘extreme persisters’ that are intolerant to disturbance due to their largest body sizes, long generation times, and low reproduction rate (they produce few, large eggs), and therefore reached higher abundances in stable and mature ecosystems [30]. DeDeyn [37] stated that changes in plant communities and biomass production did not affect the abundance of nematodes of higher trophic groups such as predators and omnivores. Our results revealed that HMG plots had numbers of both omnivores and predators similar to those in UNV plots during the whole study. Similarly, invasion of *A. syriaca* in grasslands [56], *H. sosnowskyi* in abandoned lands, forest edge, and roadside grassland habitats [37], *B. tectorum* in grasslands [53] did not affect omnivorous or predator numbers. In contrast, invasive *F. japonica* [45,63] in forests, grasslands, and wetland habitats or *S. alterniflora* [54] reduced the number of omnivores in plots where invasion had taken place. Why omnivores, or predators in some cases (habitats), react to non-native plant invasion as a typical K-strategist but not in others, remains questionable. This may be due to their diverse and often unknown feeding strategies and biology, which hampers data interpretation, species specific composition, environmental conditions, and the various density of invasive species in habitats where invasion takes place as well as the biological traits of different invasive plants [34].

## 4. Materials and Methods

### 4.1. Study Area and Study Plots

The impact of *H. mantegazzianum*, an invasive plant, on the structure of soil nematode communities was assessed in the eastern part of the Slovak Republic, village of Lekárovce (48°36′29–58″ N, 22°08′15–24″ E, 106), where *Hm* colonized about 2000 m^2^ of adjacent meadows of riverbanks of the Uh River (http://maps.sopsr.sk/mapy/invazne.php, accessed on 4 February 2021). Such an area was sufficient to define several permanent research plots. Additionally, the area colonized by *Hm* is located in the outskirts of the village, so *Hm* has never been managed by humans. Therefore, we assumed that its impact on the ecosystem did not change during its presence in the ecosystem. Estimated time of *Hm* invasion in this locality was more than 30 years. From the climatic point of view, the sampling area was in a region with a warm and slightly dry summer and cold winter. Annual temperature was 9 °C and during the vegetation/growing season, the temperature reached up to 16–17 °C. The dominant soil type is fluvisol and pseudogleys. In terms of soil reaction, these are neutral to medium acid soils.

Visually homogenous, five permanent research plots (100 m^2^) were established in both invaded (HMG) and uninvaded (UNV) areas. The HMG plots were selected with respect to the presence of a minimum of one *Hm* individual to square meter while the UNV areas had to grow only native plants. The distance between permanent plots was established as 200 m, while the distance between the invaded and uninvaded plots was established as 50 m. Such a distance was used to exclude possible water and nutrient fluxes between the study’s invaded and uninvaded plots.

The fixed phytosociological relevé method was used for the analysis of the understory plant community. Each of the five quadrats (1 m × 1 m) represented one frequency square. The vegetation was identified using a modified Braun–Blanquet abundance scale [64] without their removal from the place. The entire dataset thus contained 10 relevés for each sampling date.

### 4.2. Soil Sampling, Nematode Isolation, and Identification

Considering the natural seasonal fluctuations in nematode communities due to the variation of abiotic factors as well as vegetation development within and/or between seasons [65,66,67], the plots were sampled in May (spring), July (summer), and September (autumn) in 2017 and 2018. This allowed us to assess whether the influence of *Hm* invasion on the structure of soil nematode communities may change and/or is similar during vegetation. As nematodes are not uniformly distributed in the soil and many soil characteristics are aggregated spatially [68], soil samples were collected using a systematic design [69]. Soil samples were collected using a special garden spade. On each of the plots, ten sub-samples to the depth of 20 cm from the root rhizosphere were collected along two independent diagonal transects. Six subsamples were collected from transect 1 and four subsamples were collected from transect 2 with a random starting point. The subsamples from both transects were mixed to form one composite soil sample. A total of 60 composite samples (ten plots (five invaded and five non-invaded) × six sampling dates) were obtained. Each soil sample was separated in a zip-lock plastic bag, transferred to the laboratory and kept at 5 °C until further processing.

Nematodes were isolated by a combination of Cobb sieving and decanting [70], followed by the modified Baermann technique [71] as described by [72]. Extracted nematodes in water suspension were heat-killed, fixed, and counted under a stereomicroscope (LEICA S8APO, Germany, magnification up to 80×). At least 100 nematodes randomly selected were identified to the species level based on their morphological characteristics and morphometrics described in the original species descriptions using an Eclipse 90i light microscope (Nikon, Japan; magnifications of 100, 200, 400, 600, and 1000×). Nematode abundance was expressed as a number of individuals/100 g dry soil.

Basic physico-chemical soil parameters were simultaneously examined separately for each soil sample used for nematode analysis. Soil–moisture content was measured from fresh soil gravimetrically by oven-drying to a constant weight at 105 °C overnight. The total organic C and N were measured by using a Vario MACRO Elemental Analyzer (CNS Version; Elementar, Hanau, Germany). Soil pH was estimated potentiometrically in 1 M KCl suspension and distilled water using a digital pH meter. All study soil properties were measured as co-variables.

### 4.3. Nematode Community Analysis

The number of nematode species, nematode abundance, abundance of nematodes per trophic group, and a species diversity index [73] were evaluated. Nematode species were partitioned to several trophic groups (i.e., bacterivores, fungivores, plant parasites, root-fungal feeders, predators and omnivores [29,74]). Several maturity indices were calculated as measures of functional diversity in *Hm* invaded and uninvaded plots, the maturity index (MI, without plant parasitic nematodes), the plant parasitic index (PPI, only plant parasitic nematodes), the maturity index MI2-5 (free living c-p2 to c-p5 nematodes), and the sum maturity index *∑*MI for all nematodes. To calculate these indices, nematode species are allocated to the colonizer (c)–persister (p) scale based on their perceived life history strategy [30,75].

According to the weighted faunal analysis concept [21], the enrichment index (EI = 100 × (e/(e + b))), structure index (SI = 100 × (s/(s + b))), and channel index (CI = 0.8 Fu_2_/(3.2 Ba_1_ + 0.8 Fu_2_)) were calculated. In the faunal profile, the enrichment and structure trajectories are calculated independently from the weighted abundance of nematodes in guilds representing basal (*b*), enrichment (*e*), and structure (*s*) food web components. For example, the *b* component is calculated as *kbnb*, where *kb* is the weighting assigned to the guilds, which indicates the basal characteristics of the food web (Ba2, Fu2), and *nb* is the abundances of nematodes in these guilds. The *e* and *s* components can be calculated similarly using the guilds indicating enrichment (Ba1, Fu2) and structure (Ba3–Ba5, Fu3–Fu5, Om3–Om5, Ca2–Ca5), respectively. The EI indicates basal/enriched while the SI indicates structured/stable soil food web conditions. CI is an indicator of fungal-mediated dominance of organic-matter decomposition. A high CI (>50%) indicates a higher proportion of fungal decomposition. A low CI (<50%) suggests bacterial decomposition channels [31]. Following Ferris et al. [76], several metabolic footprints (composite, enrichment, structure, herbivore, fungivore, bacterivore, predator, omnivore footprint) have also been calculated. These footprints provide the metrics for the magnitudes of ecosystem functions and services provided by component organisms of the soil food web.

### 4.4. Data Analysis

The statistical analyses were performed separately for each sampling date (May, June, September) of two vegetation seasons (2017, 2018) and data were compared between the HMG plots and the UNV as control plots (*n* = 10). All nematological data including the indices, metabolic footprints as well as the soil physico-chemical properties were calculated as the means for the individual plots and sampling dates of the two investigated seasons and compared using Tukey’s honestly significant difference (HSD) post-hoc test (*p* < 0.05; *p* < 0.01). Factorial analysis of variance (two-way ANOVA) was used to exam the main and interaction effects of sampling date, season, and invasion status on the nematode abundance, trophic groups, and soil physico-chemical properties. Nonparametric Spearman’s correlation coefficient (rs) was calculated to test the relationships between the nematode community parameters and soil physicochemical properties for each sample. Correlations obtained at *p* < 0.05, *p* < 0.01, and *p* < 0.001 were considered significant. The data were log-transformed before analysis to improve normality. Statistical analyses were performed using the PlotIT (Statistical Software Vers. 3.2, Scientific Programming Enterprises, Haslett, MI, USA).

Co-correspondence analysis (Co-CA) of the plant communities with the nematode trophic groups and nematode abundance was performed as a single step to determine the effect of changes in the plant community due to the invasion of *H. mantegazzianum* on the native plant species and structure of the nematode communities [77]. The single-step approach rendered the Co-CA superior to a canonical correspondence analysis because the number of predictors exceeded the number of plots (seven nematode community parameters, 24 plant species, 10 co-located plots) by an order of magnitude [77]. Our approach was modeled after using Co-CA to investigate the association between the plant and nematode communities [34,78]. All multivariate analyses were performed using CANOCO version 5 (Version 5.04) Microcomputer Power, Ithaca, NY [79]. Community indices and metabolic footprints were calculated using the NINJA online program [80] (https://sieriebriennikov.shinyapps.io/ninja/, accessed on 21 June 2014).

## 5. Conclusions

Soils are the most biologically diverse and thus the most threatened environments in terms of biodiversity loss. Many threats such as soil erosion, land use change, overexploitation, and pollution including biological invasion have been identified as directly disturbing soil organism abundance, distribution, and activity. However, relatively few studies have investigated the plant invasion impacts on the biological diverse and abundant soil nematodes. Therefore, we sought to understand how invasive *H. mantegazzianum* alters the nematode communities considering changes in native plant species and soil properties. Hogweed invasion increased nematode abundance, number of bacterivores and plant parasites, and decreased abundance of omnivores, while total nematode biomass and species diversity were not affected by invasion. Moreover, we sought to compare whether invasive *H mantegazzianum* influenced the nematode communities similar to botanically related species *H. sosnowskyi* from our previous studies. Here, the analysis of soil nematode communities in plots long-term invaded by *Hm* compared with equivalent non-invaded control plots showed different answers than those found in related *H. sosnowskyi*, while some community parameters were affected by the same pattern. For example, *H. sosnowskyi* invasion decreased nematode abundance, species diversity, number of plant parasites, increased and/or not affected bacterivore abundance, while it decreased omnivore abundance. Our findings thus suggest that two closely related species may have various impacts on soil biota. The likely reason for this is are the various habitat characteristics and native plant species compositions able to grow with invader or specific nematode species composition in ecosystems where invasion takes place.

## Figures and Tables

**Table 1:** Soil physico-chemical properties (mean ± S.D.) of the investigated plots of *Heracleum mantegazzianum* invaded and uninvaded control plots in three vegetation seasons and the two years of 2017 and 2018.

	Spring (May)		Summer (July)		Autumn (September)	
	HMG	UNV	HMG	UNV	HMG	UNV
SM	16.15 ± 2.88	12.82 ± 1.58	25.45 ± 2.11	26.78 ± 2.61	19.69 ± 1.28	18.93 ± 2.04
pH (KCl)	7.20 ± 0.10 *	6.23 ± 0.28	7.02 ± 0.12 *	6.11 ± 0.12	6.75 ± 0.24 *	6.00 ± 0.12
N_tot_	0.22 ± 0.01 *	0.31 ± 0.02	0.23 ± 0.01	0.22 ± 0.02	0.24 ± 0.03	0.25 ± 0.03
C_ox_	1.81 ± 0.10 *	2.38 ± 0.11	2.00 ± 0.07 *	2.45 ± 0.18	1.92 ± 0.01	1.78 ± 0.20
C/N	7.90 ± 0.25	7.89 ± 0.36	8.25 ± 0.30	9.76 ± 0.78	8.19 ± 0.53	7.49 ± 0.69

Different from uninvaded control according the Tukey’s (HSD) post-hoc test (* for *p* < 0.05) (*n* = 10). HMG—*Heracleum mantegazzianum* invaded plots*;* UNV—uninvaded control; SM—soil moisture (% of initial weight); pH (KCl)—soil acidity; N_tot_—soil nitrogen content (% of dry weight); C_ox_—soil carbon content (% of dry weight); C/N—ratio of carbon to nitrogen.

**Table 2:** Mean percentage of plant cover in the *Heracleum mantegazzianum* invaded and uninvaded control plots in the three vegetation seasons and two years (2017, 2018) (*n* = 10).

Plants	Invaded			Uninvaded Control		
	Spring	Summer	Autumn	Spring	Summer	Autumn
*Achillea milleofolium*	-	-	-	1.5	1	2.6
*Dactylis glomerata*	-	-	-	18.1	10.9	6.5
*Elymus repens*	1.7	1.1	5.4	-	-	-
*Festuca* sp.	-	-	-	21.5	26	17
*Fragaria vesca*	-	-	-	7.8	5.5	2.5
*Galium odoratum*	8.9	7.5	4.7	-	0.5	2.2
*Heliantus tuberosus*	3.8	16	22.3	-	-	-
*Heracleum mantegazzianum*	83	80.1	53.9	-	-	-
*Lathyrum pratensis*	-	-	-	1.7	0.5	0.3
*Leucanthemum vulgare*	-	-	-	0.9	0.1	-
*Marticaria chamomilla*	-	-	-	0.4	1.2	0.5
*Medicago* sp.	-	-	-	5.2	1.1	3.9
*Poa pratensis*	-	-	-	2.4	1.6	2.3
*Prunella vulgaris*	-	-	-	0.3	0	0.2
*Pseudolysimachion longifolium*	-	-	-	-	0.3	0.5
*Pulmonaria officinalis*	0.2	-	-	0.3	-	-
*Ranunculus acris*	-	-	-	1.4	1.7	-
*Rumex acetosella*	0.1	-	0.1	1.4	2.5	2.4
*Silene vulgaris*	-	-	-	4.6	4	0.5
*Silybum marianum*	5.5	6	12.4	1.9	0.4	0.9
*Trifolium repens*	-	-	-	7.6	8.2	5.1
*Trisetum flavescens*	-	-	-	10.5	14	8.2
*Urctica dioica*	13.1	20.8	18.7	0.3	0.9	0.4
*Vicia sativa*	-	-	-	2.2	1.2	0.6

**Table 3:** Mean abundance of nematode species (100 g/dry soil) of investigated plots of *Heracleum mantegazzianum* invaded and uninvaded control plots in the three vegetation seasons and two years 2017, 2018 (*n* = 10).

		Spring		Summer		Autumn	
	c-p	HMG	UNV	HMG	UNV	HMG	UNV
**Bacterivores**							
*Acrobeles ciliatus*	2	-	0.8	0.6	0.5	-	-
*Acrobeloides nanus*	2	6.0	2.7	6.9	8.1	8.7	10.4
*Alaimus parvus*	4	2.3	0.2	6.7	1.7	4.5	0.4
*Alaimus primitivus*	4	33.8	4.0	30.1	7.7	24.4	6.7
*Alaimus robustus*	4	7.4	1.7	8.5	-	3.2	0.5
*Amphidelus coronatus*	4	2.1	-	-	-	0.3	-
*Aulolaimus oxycephalus*	3	-	0.6	-	0.6	-	-
*Cephalobus parvus*	2	0.7	-	-	-	2.0	1.5
*Cephalobus persegnis*	2	5.2	3.3	21.6	4.9	16.9	1.7
*Cervidellus vexilliger*	2	-	0.5	0.6	1.2	0.2	0.2
*Drilocephalobus coomansi*	2	-	-	-	-	-	-
*Eucephalobus mucronatus*	2	14.2	8.6	13.7	1.5	9.0	5.4
*Eucephalobus oxyuroides*	2	2.6	0.3	1.0	0.8	2.7	1.3
*Eucephalobus striatus*	2	18.6	7.1	31.7	7.3	13.1	7.5
*Heterocephalobus elongatus*	2	-	-	-	0.2	-	0.4
*Heterocephalobus eurystoma*	2	1.6	-	1.0	1.4	3.5	4.2
*Chiloplacus propinquus*	2	0.5	-	2.3	2.1	3.8	2.5
*Mesorhabditis labiata*	1	4.0	-	0.9	0.8	3.2	1.8
*Panagrolaimus ridigus*	1	2.8	-	11.6	0.8	1.5	0.3
*Paramphidelus dolichurus*	4	4.0	-	0.1	-	1.0	-
*Plectus longicaudatus*	2	0.6	1.3	-	1.3	-	-
*Plectus opisthocirculus*	2	-	-	-	-	0.3	0.4
*Plectus parietinus*	2	0.6	0.5	-	-	0.7	0.1
*Plectus parvus*	2	-	-	2.6	14.4	-	-
*Prismatolaimus intermedius*	3	1.4	0.2	-	1.6	0.7	0.4
*Protorhabditis filiformis*	1	-	-	-	-	0.6	-
*Rhabditis* spp. juvs.	1	16.2	8.6	27.6	7.9	18.1	3.6
**Fungivores**							
*Aphelenchoides parietinus*	2	18.3	1.8	4.6	0.4	5.9	0.9
*Aphelenchus avenae*	2	8.0	3.8	11.7	1.9	12.0	2.5
*Diptherophora communis*	3	23.1	10.6	14.1	16.9	18.8	16.3
*Ditylenchus intermedius*	2	3.2	3.6	3.4	3.6	5.9	2.5
*Ditylenchus longimetricalis*	2	-	-	1.2	-	-	-
*Tylencholaimellus striatus*	4	0.6	1.7	-	0.6	-	1.5
*Tylencholaimus minimus*	4	1.4	2.6	-	8.2	-	-
*Tylencholaimus stecki*	4	-	1.7	0.8	11.8	3.4	4.3
**Plant parasites**							
*Bitylenchus dubius*	3	2.1	0.6	-	-	2.8	-
*Criconemoides informis*	3	4.5	11.9	2.0	18.1	-	-
*Geocenamus brevidens*	3	16.0	5.8	13.6	1.6	6.5	8.2
*Geocenamus microdorus*	3	7.3	0.7	10.5	4.0	15.7	2.7
*Geocenamus nanus*	3	5.5	-	5.6	0.6	2.3	-
*Gracilacus straeleni*	2	-	-	-	0.4	-	0.5
*Helicotylenchus canadensis*	3	2.2	10.1	3.5	1.8	4.0	4.8
*Helicotylenchus digonicus*	3	11.5	34.8	14.0	10.4	18.5	30.8
*Longidorus elongatus*	5	-	0.4	-	1.3	-	5.5
*Meloidogyne* sp.	3	1.2	1.2	2.0	6.0	3.0	1.4
*Mesocriconema curvatum*	3	-	0.6	-	-	-	-
*Paratylenchus microdorus*	2	72.6	0.5	30.6	1.5	59.4	0.4
*Paratylenchus projectus*	2	10.3	5.3	8.8	2.8	18.8	11.5
*Pratylenchoides crenicauda*	3	3.3	0.5	1.5	1.1	1.9	2.5
*Pratylenchus neglectus*	3	2.1	2.9	-	-	-	3.1
*Pratylenchus pratensis*	3	1.8	4.4	8.1	4.0	3.3	1.8
*Pratylenchus thornei*	3	-	3.7	0.8	0.8	0.3	5.7
*Xiphinema simile*	5	-	-	-	2.5	-	0.5
**Root-fungal feeders**							
*Boleodorus thylactus*	2	2.9	5.1	8.3	0.2	2.6	2.8
*Basiria gracilis*	2	-	0.1	2.1	-	-	0.6
*Coslenchus costatus*	2	4.4	3.6	11.2	4.4	10.5	16.5
*Filenchus discrepans*	2	0.4	-	0.5	0.8	1.7	1.4
*Filenchus thornei*	2	1.1	0.6	-	-	0.4	5.0
*Filenchus vulgaris*	2	4.3	15.3	7.4	24.6	14.6	20.3
*Malenchus exiguus*	2	3.8	2.6	1.3	1.8	0.8	2.0
*Psilenchus hilarulus*	2	2.3	0.9	0.3	8.1	0.9	0.8
*Tylenchus elegans*	2	-	1.7	-	2.0	-	3.0
**Omnivores**							
*Aporcelaimellus obtusicaudatus*	4	13.2	10.2	9.7	19.4	3.4	6.1
*Campydora demonstrans*	4	1.1	4.1	-	0.2	2.8	1.2
*Dorylaimoides mickoletskyi*	4	-	1.8	0.6	1.8	0.3	1.5
*Dorylaimus bryophilus*	4	11.9	13.3	19.4	26.4	10.0	11.2
*Dorylaimus microdorus*	4	1.4	-	0.3	1.3	0.3	0.2
*Ecumenicus monohystera*	4	0.8	2.4	4.1	0.2	2.8	0.4
*Eudorylaimus acuticauda*	4	3.4	0.4	0.4	3.4	-	0.7
*Eudorylaimus similis*	4	-	-	1.2	7.2	0.3	1.2
*Mesodorylaimus bastiani*	5	0.4	2.8	3.0	-	-	-
*Microdorylaimus parvus*	4	7.2	2.6	6.0	3.7	4.2	2.0
*Paraxonchium laetificans*	5	-	1.9	-	0.2	-	0.2
*Prodorylaimus brigdamensis*	5	0.7	-	1.8	3.8	2.7	5.5
*Pungentus engadinensis*	4	-	1.4	0.7	2.4	0.9	1.7
*Pungentus silvestris*	4	-	10.7	-	3.4	-	-
*Thonus ettersbersgensis*	4	0.4	0.7	0.3	1.4	0.2	1.7
**Predators**							
*Clarkus papillatus*	4	2.5	1.0	0.3	2.8	0.3	1.1
*Discolaimoides bulbiferus*	5	10.4	2.7	5.3	2.4	10.2	4.2
*Discolaimus major*	5	0.8	2.2	0.8	0.6	-	0.9
*Enchondelus macrodorus*	4	1.1	-	-	-	0.9	-
*Mylonchulus brachyuris*	4	7.4	2.0	5.4	2.0	2.9	1.3
*Oxydirus oxycephalus*	5	0.2	0.8	-	0.2	1.1	0.7
*Trypila filicaudata*	3	-	-	-	3.4	-	-

HMG—*Heracleum mantegazzianum* invaded plots; UNV—uninvaded control

**Table 4:** Mean values (±S.D.) for nematode abundance, species number, trophic groups, and community descriptors of investigated plots of *Heracleum mantegazzianum* invaded and uninvaded control plots in the three vegetation seasons and two years of 2017 and 2018.

	Spring (May)		Summer (July)		Autumn (September)	
	HMG	UNV	HMG	UNV	HMG	UNV
Abundance (100 g/dry soil)	401.5 ± 174.2 *	245.5 ± 59.9	412 ± 200.2	288.9 ± 150.5	375.4 ± 100.6	250.3 ± 54.2
Nematode species number	42.2 ± 3.6	38.7 ± 4.1	36 ± 3.1 *	45 ± 2.5	36 ± 2.4 *	43 ± 0.8
Bacterivores	124.6 ± 53.2 *	39.8 ± 12.8	170.6 ± 100.2 *	61.8 ± 30.7	115.8 ± 30.6 *	52.5 ± 13.2
Fungivores	55.6 ± 15.9 *	26.5 ± 10.1	34.5 ± 19.2	43.6 ± 20.3	46.5 ± 20.8	33.9 ± 15.4
Omnivores	42.5 ± 30.6	54.1 ± 38.7	39.8 ± 40.6	80.1 ± 23.6	25.4 ± 20.4	35.8 ± 10.1
Predators	20.1 ± 10.9 **	2.8 ± 2.0	10.9 ± 11.5	10.3 ± 6.6	13.8 ± 8.4	7.9 ± 3.8
Root-fungal feeders	18.9 ± 16.5	29.5 ± 20.9	36.8 ± 21.8	40.1 ± 30.7	30.1 ± 15.6	49.7 ± 13.4
Plant parasites	139.7 ± 55.8	89.9 ± 48.7	97.7 ± 48.3	50.2 ± 35.7	135.6 ± 74.5	82.7 ± 27.6
Species diversity index	2.63 ± 0.23	2.86 ± 0.20	2.83 ± 0.10	2.79 ± 2.68	2.75 ± 0.32	3.03 ± 0.12
Maturity index	2.95 ± 0.48	2.91 ± 0.57	2.65 ± 0.17 *	3.11 ± 0.26	2.76 ± 0.03 *	2.91 ± 0.14
Maturity index (2–5)	3.15 ± 0.44	3.12 ± 0.40	2.95 ± 0.28 *	3.39 ± 0.07	2.95 ± 0.25 *	3.25 ± 0.08
Sum Maturity index	2.70 ± 0.35	2.79 ± 0.42	2.65 ± 0.11 *	3.17 ± 0.21	2.62 ± 0.13	2.86 ± 0.15
Plant parasitic index	2.40 ± 0.24 *	2.75 ± 0.10	2.66 ± 0.09 *	3.04 ± 0.21	2.45 ± 0.18 *	2.89 ± 0.12
Channel index	30.32 ± 15.18 *	50.11 ± 11.28	18.18 ± 8.33 *	42.81 ± 18.19	35.97 ± 15.45 *	66.81 ± 17.2
Enrichment index	53.24 ± 17.26	54.35 ± 18.84	61.81 ± 11.15	52.69 ± 20.37	53.31 ± 10.8	44.44 ± 12.83
Structure index	80.96 ± 15.42	79.52 ± 17.13	77.13 ± 9.15	87.21 ± 6.36	77.75 ± 11.8	80.34 ± 5.82
Total nematode biomass	0.95 ± 0.84	0.85 ± 0.0.72	1.37 ± 0.85	1.72 ± 0.64	0.73 ± 0.34	0.75 ± 0.57
Composite footprint	175.1 ± 127.2	145.9 ± 66.8	228.3 ± 181.9	259.5 ± 261.7	140.8 ± 70.7	128.7 ± 78.3
Enrichment footprint	36.2 ± 13.2 *	19.8 ± 11.6	60.2 ± 40.5 *	19.5 ± 12.8	40.7 ± 25.7 *	10.8 ± 7.8
Structure footprint	118.9 ± 116.4	108.8 ± 39.2	142.3 ± 100.5	222.1 ± 189.5	70.8 ± 64.7	94.5 ± 37.8
Herbivore footprint	13.6 ± 4.8	14.7 ± 10.8	15.8 ± 9.6	11.8 ± 6.5	20.7 ± 24.6	23.8 ± 15.1
Fungivore footprint	4.9 ± 2.7	5.1 ± 2.3	4.9 ± 3.6	8.7 ± 5.4	7.4 ± 1.7	6.7 ± 3.1
Bacterivore footprint	45.8 ± 39.7	18.6 ± 10.8	70.9 ± 30.5 **	22.5 ± 11.8	45.7 ± 21.1 *	12.1 ± 6.6
Predator footprint	5.8 ± 3.6	3.7 ± 3.8	4.4 ± 5.1	5.1 ± 5.6	4.9 ± 2.1	3.6 ± 2.8
Omnivore footprint	100.3 ± 27.9	97.5 ± 50.1	130.3 ± 68.7	215.1 ± 257.8	62.7 ± 43.3	74.4 ± 62.8

Different from uninvaded control according the Tukey’s (HSD) post-hoc test (* for *p* < 0.05; ** for *p* < 0.01) (*n* = 10); HMG- *Heracleum mantegazzianum* invaded plots; UNV—uninvaded control.

## Supplementary Materials

The following are available online at https://www.mdpi.com/article/10.3390/plants10102103/s1. Table S1: Factorial analysis of variance. Effect of three factors (season, year and invasion status) on the physico-chemical soil parameters, Table S2: Searman’s rank correlation between nematode abundance, species number, nematode trophic groups, ecological indices and soil properties, Table S3: Factorial analysis of variance. Effect of three factors (season, year and invasion status) on abundance of nematodes within particular nematode trophic groups.
