# Supplementary material for: Long-Term Giant Hogweed Invasion Contributes to the Structural Changes of Soil Nematofauna
Source: Plants (Basel). 2021 Oct 4;10(10):2103. doi: 10.3390/plants10102103 (PMC8541641; doi:10.3390/plants10102103)
Supplement: Supplementary file 1 [file plants-10-02103-s001.zip › plants-1387636-supplementary.pdf]

**Table S1** Factorial analysis of variance. Effect of three factors (season, year and invasion status) on the physico-chemical soil parameters

| Factors | Degrees of freedom | Sum of squares | Mean squares | F values | Significant |
|---|---|---|---|---|---|
| Factor A (C) | 2 | 0.009 | 0.005 | 6.9201 | ** |
| Factor B | 1 | 0.001 | 0.001 | 1.4628 | - |
| Factor C | 1 | 0.007 | 0.004 | 10.5180 | ** |
| A x B | 2 | 0.000 | 0.000 | 0.2059 | - |
| A x C | 2 | 0.019 | 0.009 | 14.2652 | ** |
| B x C | 1 | 0.000 | 0.000 | 0.4259 | - |
| A x B x C | 2 | 0.000 | 0.000 | 0.3676 | - |
| Factor A (N) | 2 | 0.883 | 0.441 | 4.9203 | * |
| Factor B | 1 | 0.023 | 0.023 | 0.2531 | - |
| Factor C | 1 | 0.706 | 0.706 | 7.8731 | ** |
| A x B | 2 | 0.028 | 0.017 | 0.1535 | - |
| A x C | 2 | 0.484 | 0.242 | 2.6981 | * |
| B x C | 1 | 0.024 | 0.024 | 02645 | - |
| A x B x C | 2 | 0.028 | 0.014 | 0.1549 | - |
| Factor A (C/N) | 2 | 39.369 | 19.384 | 33.6590 | ** |
| Factor B | 1 | 0.003 | 0.003 | 0.0049 | - |
| Factor C | 1 | 3.752 | 3.752 | 6.4157 | * |
| A x B | 2 | 0.000 | 0.000 | 0.003 | - |
| A x C | 2 | 19.359 | 9.679 | 16.5510 | ** |
| B x C | 1 | 0.001 | 0.001 | 0.0009 | - |
| A x B x C | 2 | 0.002 | 0.001 | 0.0013 | - |
| Factor A (pH) | 2 | 1.086 | 0.543 | 274.700 | ** |
| Factor B | 1 | 0.000 | 0.000 | 0.2053 | - |
| Factor C | 1 | 11.530 | 11.530 | 5835.57 | ** |
| A x B | 2 | 0.000 | 0.000 | 0.1212 | - |
| A x C | 2 | 0.274 | 0.137 | 69.2934 | ** |
| B x C | 1 | 0.001 | 0.001 | 0.7094 | - |
| A x B x C | 2 | 0.001 | 0.000 | 0.2492 | - |
| Factor A (SM) | 2 | 1476.941 | 738.470 | 1736.8896 | ** |
| Factor B | 1 | 0.000 | 0.000 | 0.0011 | - |
| Factor C | 1 | 14.910 | 14.910 | 35.0688 | ** |
| A x B | 2 | 0.013 | 0.007 | 0.0154 | - |
| A x C | 2 | 38.666 | 19.333 | 45.4716 | ** |
| B x C | 1 | 0.439 | 0.439 | 1.0316 | - |
| A x B x C | 2 | 0.656 | 0.328 | 0.7716 | - |

Factor A (year); Factor B (season); Factor C (invasion status) ** F values significant at $P< 0.01$; * F values significant at $P<0.05$. SM, soil moisture (% of initial weight); pH ($H_2O$), soil acidity; N, soil nitrogen content (% of dry weight); C, soil carbon content (% of dry weight); C:N, ratio of carbon to nitrogen

**Table S2.** Spearman´s rank correlation between nematode abundance, species number, nematode trophic groups, ecological indices and soil properties

| | SM | pH (KCl) | N | C | C/N |
|---|---|---|---|---|---|
| Nematode abundance | ns | -0.3951** | ns | ns | ns |
| Nematode species number | ns | -0.2692* | ns | ns | ns |
| Bacterivores | ns | ns | ns | ns | ns |
| Fungivores | ns | ns | 0.2863* | ns | ns |
| Omnivores | 0.2514* | ns | ns | 0.3438** | ns |
| Predators | ns | ns | ns | ns | ns |
| Plant Parasites | 0.3169* | -0.2887* | ns | ns | ns |
| Root-fungal feeders | ns | -0.4426** | ns | ns | ns |
| Species diversity index | ns | ns | ns | ns | ns |
| Maturity index | ns | ns | -0.2479* | 0.3554* | ns |
| Maturity index (2-5) | ns | ns | ns | ns | ns |
| Sum Maturity index | ns | ns | -0.3127** | ns | ns |
| Plant parasite index | ns | -0.526*** | ns | ns | ns |
| Enrichment index | ns | ns | ns | ns | ns |
| Structure index | ns | ns | ns | ns | ns |
| Channel index | ns | ns | ns | ns | ns |
| Total nematode biomass | ns | -0.2556* | 0.4598** | 0.2642* | ns |

SM. soil moisture (% of initial weight); pH (KCl). soil acidity; N. soil nitrogen content (% of dry weight); C. soil carbon content (% of dry weight); C:N. ratio of carbon to nitrogen. * = P < 0.05; ** = P < 0.01; *** = P < 0.001; ns = not significant.

**Table S3** Factorial analysis of variance. Effect of three factors (season, year and invasion status) on abundance of nematodes within particular nematode trophic groups.

| Factors | Degrees of freedom | Sum of squares | Mean squares | F values | Significant |
|---|---|---|---|---|---|
| Factor A (Bacterivores) | 2 | 13015.84 | 6507.92 | 0.5771 | - |
| Factor B | 1 | 57010.83 | 57010.83 | 5.0555 | * |
| Factor C | 1 | 631999.62 | 63199.62 | 5.6043 | * |
| A x B | 2 | 24257.85 | 12128.92 | 1.0755 | - |
| A x C | 2 | 16946.35 | 8473.17 | 0.7514 | - |
| B x C | 1 | 1004.50 | 1004.50 | 0.0891 | - |
| A x B x C | 2 | 7432.86 | 3716.43 | 0.3296 | - |
| Factor A (Fungivores) | 2 | 83.258 | 41.629 | 0.0369 | - |
| Factor B | 1 | 1847.04 | 1847.04 | 1.6358 | - |
| Factor C | 1 | 1846.83 | 1846.83 | 1.6516 | - |
| A x B | 2 | 2474.48 | 1373.74 | 1.1267 | - |
| A x C | 2 | 3424.69 | 1712.34 | 1.5166 | - |
| B x C | 1 | 2949.80 | 2949.80 | 2.6125 | - |
| A x B x C | 2 | 301.90 | 150.95 | 0.1337 | - |
| Factor A (Omnivores) | 2 | 9699.47 | 4849.73 | 2.9330 | * |
| Factor B | 1 | 23510.52 | 23510.52 | 14.2184 | ** |
| Factor C | 1 | 2896.76 | 2896.76 | 1.7519 | - |
| A x B | 2 | 5578.85 | 2789.42 | 1.6870 | - |
| A x C | 2 | 1412.336 | 706.16 | 0.4271 | - |
| B x C | 1 | 6400.46 | 6400.48 | 3.8708 | * |
| A x B x C | 2 | 2917.00 | 1458.50 | 0.8821 | - |
| Factor A (Predators) | 2 | 1018.933 | 509.46 | 3.0473 | * |
| Factor B | 1 | 2710.84 | 271.84 | 16.2145 | ** |
| Factor C | 1 | 357.70 | 357.70 | 2.1396 | - |
| A x B | 2 | 3089.43 | 1544.71 | 9.2395 | ** |
| A x C | 2 | 289.55 | 144.78 | 0.8660 | - |
| B x C | 1 | 590.94 | 590.94 | 3.5347 | - |
| A x B x C | 2 | 1022.75 | 511.37 | 3.0587 | - |
| Factor A (Root-fungal feed) | 2 | 10305.84 | 5152.92 | 1.2937 | - |
| Factor B | 1 | 2509.06 | 2509.06 | 0.6299 | - |
| Factor C | 1 | 44542.85 | 44542.85 | 11.1830 | ** |
| A x B | 2 | 23592.35 | 11796.17 | 2.9616 | - |
| A x C | 2 | 224.108 | 112.054 | 0.0281 | - |
| B x C | 1 | 4590.41 | 4950.41 | 1.2428 | - |
| A x B x C | 2 | 7716.57 | 3858.28 | 0.9687 | - |
| Factor A (Plant parasites) | 2 | 3242.03 | 1621.01 | 2.2007 | - |
| Factor B | 1 | 1571.84 | 1571.84 | 2.1340 | - |
| Factor C | 1 | 2732.40 | 2732.40 | 3.7096 | * |
| A x B | 2 | 1049.45 | 524.27 | 0.7124 | - |
| A x C | 2 | 303.85 | 151.92 | 0.2063 | - |
| B x C | 1 | 6931.60 | 6931.60 | 9.4125 | ** |
| A x B x C | 2 | 5015.96 | 2507.98 | 3.4049 | * |

Factor A (year); Factor B (season); Factor C (invasion status) ** F values significant at $P< 0.01$;
* F values significant at $P<0.05$
